# Effect of Benzothiadiazole-Based π-Spacers on Fine-Tuning of Optoelectronic Properties of Oligothiophene-Core Donor Materials for Efficient Organic Solar Cells: A DFT Study

**DOI:** 10.1021/acs.jpca.3c04866

**Published:** 2023-12-12

**Authors:** Rania Zaier, Arnaud Martel, Tomasz J. Antosiewicz

**Affiliations:** † Faculty of Physics, University of Warsaw, Pasteura 5, PL-02-093 Warsaw, Poland; ‡ Institut des Molécules et Matériaux du Mans, UMR 6283 CNRS-Université du Maine, Avenue Olivier Messiaen, 72085 Cedex Le Mans, France

## Abstract

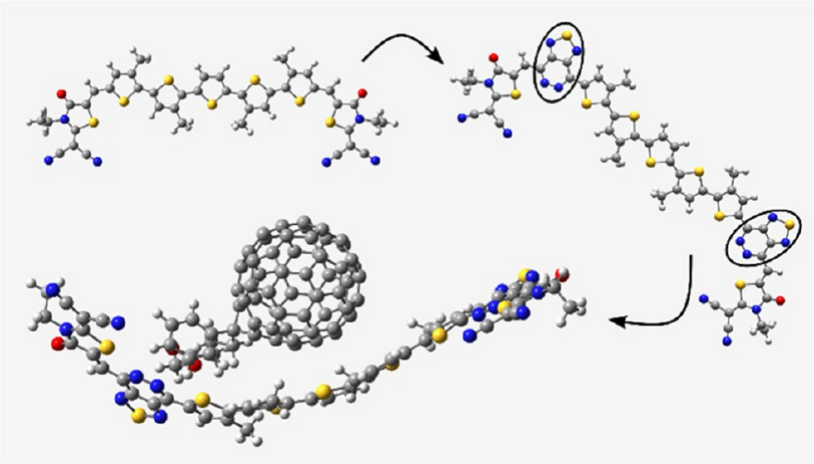

In this work, five novel A-π-D-π-A type molecules D1–D5 were designed by adding unusual benzothiadiazole derivatives as π-spacer blocks to the efficient reference molecule DRCN5T for application as donor materials in organic solar cells (OSCs). Based on a density functional theory approach, a comprehensive theoretical study was performed with different functionals (B3LYP, B3LYP-GD3, B3LYP-GD3BJ, CAM-B3LYP, M06, M062X, and wB97XD) and with different solvent types (PCM and SMD) at the extended basis set 6-311+g(d,p) to evaluate the structural, optoelectronic, and intramolecular charge transfer properties of these molecules. The B3LYP-GD3BJ hybrid functional was used to optimize the studied molecules in CHCl_3_ solution with the SMD model solvent as it provided the best results compared to experimental data. Transition density matrix maps were simulated to examine the hole–electron localization and the electronic excitation processes in the excited state, and photovoltaic parameters including open-circuit photovoltage and fill factor were investigated to predict the efficiency of these materials. All the designed materials showed promising optoelectronic and photovoltaic characteristics, and for most of them, a red shift. Out of the proposed molecules, [1,2,5]thiadiazolo[3,4-*d*]pyridazine was selected as a promising π-spacer block to evaluate its interaction with PC_61_BM in a composite to understand the charge transfer between the donor and acceptor subparts. Overall, this study showed that adding π-spacer building blocks to the molecular structure is undoubtedly a potential strategy to further enhance the performance of donor materials for OSC applications.

## 1 Introduction

Nowadays, the demand for energy is increasing significantly due to human activity.^1,2^ Traditional energy resources such as fossil fuels or nuclear energy are criticized because of pollution generated during production and the danger they pose to the environment.^3^ Therefore, renewable sources of energy have emerged as a promising alternative in the last two decades thanks to several benefits at the social, environmental and economic sides as they are inexhaustible, reliable and nontoxic with the potential to improve public health and partially mitigate global warming.^4,5^

Solar energy is one of the most promising renewable alternatives to fossil and nuclear energies due to its great potential to respond to the planet’s energy needs.^6^ Photovoltaic technology has thus grown and developed in recent years. Presently this technology is mainly based on solar panels made from silicon; nevertheless, silicon modules have the disadvantages of strong dependence on weather and high cost of production.^7−9^ Hence, an alternative solution based on organic compounds, called organic photovoltaics (OPVs), has received great consideration with the hopes of enabling green technology through organic solar cell (OSC) devices for clean energy development in order to achieve environmental sustainability.^10−12^ OSCs also offer promising manufacturing characteristics over their inorganic counterparts due to roll-to-roll (R2R) processing based on flexible substrate technology.^13^ R2R fabrication affords various benefits such as increasing efficiency, enabling multiple sequential processing steps, high production yields, and reduced manufacturing costs.^14^

Recently, OSCs based on a bulk heterojunction (BHJ) architecture have gained great attention. In BHJ structures the wide extension of the contact between the donor and acceptor materials leads to a considerable increase of exciton dissociation and therefore in the power conversion efficiency (PCE) of OSCs.^15,16^ Advances of BHJ OSCs have been linked to the development of new organic materials with tremendous semiconducting and electro-optical properties. Intense research has been conducted in the search for efficient building blocks for synthesis of new polymers and small molecules (SMs) exhibiting excellent properties for OPV applications.^17^ Polymers have long been favored materials in the field of organic solar cells, primarily due to their advantageous characteristics.^18^ Their inherent structural flexibility, ease of processing, and tunable optoelectronic properties have made them compelling choices for the design and fabrication of efficient organic photovoltaic devices.^19^ Over the years, significant progress has been made in improving the energy conversion efficiency of polymer-based solar cells, making them a major player in the field of renewable energy. However, recent advancements in materials science have unveiled a compelling alternative: namely, small organic molecules. Compared to polymers, the interest in studying SMs stems from their well-defined molecular structure, higher purification, and simple synthetic procedures. Recently, small molecule donor systems have successfully enabled high PCE in OPV devices.^20^ Different conformations of small molecule donor materials such as D-A-D, D-π-A, A-D-A, and D-π-A-π-D have been investigated and shown to exhibit wide optical absorption, good film quality, and high carrier mobility in photovoltaic applications.^21−23^ Particularly, the linear conjugated architecture of the A-π-D-π-*A* type was widely used as an effective molecular design in which the central core of an electron-rich donor block (D) is covalently linked with two electron-deficient terminal acceptor blocks (A) through two π-conjugated bridges. This structure efficiently reduces the band gap energy, tunes the optoelectronic properties, and enhances the intramolecular charge transfer (ICT) among the different moieties of the donor material.^24,25^

**Figure 1:**
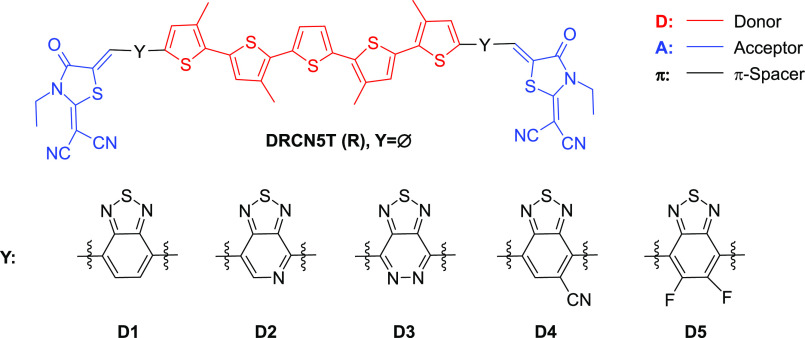
Molecular structures of the reference R molecule of DRCN5T and five specific π-spacer groups that are inserted between the donor and acceptor groups of R. The newly designed molecules are referred to by the name of the employed spacer D1–D5.

In this contribution, we designed and characterized five A-π-D-π-A SMs based on the A-D-A reference structure. The reference DRCN5T chosen in this work is characterized by high photovoltaic performance and is composed of an oligothiophene core donor block and two 2-(3-ethyl-4-oxothiazolidin-2-ylidene)malononitrile end-capped acceptor blocks.^26,27^ The designed materials are push–pull systems, where the alternating arrangement of electron-deficient and electron-rich blocks along the conjugated framework efficiently extends the electron delocalization, enhances the light-harvesting abilities, and improves the charge dissociation for efficient OPVs applications.^28,29^ The selected π-spacer for the new materials design is 2,1,3-benzothiadiazole (BT), which is one of the most popular fused heterocyclic building blocks for organic electronics thanks to its outstanding optoelectronic properties.^30^ As depicted in Figure 1, the designed materials, labeled D1–D5, contain different BT derivatives in which carbon atoms are replaced by electron-donating groups such as nitrogen atoms, or electron-withdrawing groups such as fluorine or cyano groups.^31^ Detailed computational investigations were carried out to evaluate the influence of the introduction of such modifications on the structural, electronic, and optical properties of donor materials for efficient OSC devices.

## 2 Computational Methodology

Gaussian16 software^32^ was used to perform the theoretical calculations using the density functional theory (DFT) and time-dependent density functional theory (TDDFT) approaches.^33,34^ We start by choosing the appropriate functional to reproduce the experimental data of the reference molecule (DRCN5T). The ground-state optimization of DRCN5T was performed using different exchange–correlation (XC) functionals such as the Becke-3-Lee–Yang–Parr (B3LYP) XC functional, B3LYP coupled with Grimme’s D3 atomic pairwise dispersion correction (B3LYP-GD3) to estimate noncovalent interaction, B3LYP-GD3 combined with Becke–Johnson (BJ) damping (B3LYP-GD3BJ),^35,36^ B3LYP combined with the Coulomb-attenuating method (CAM-B3LYP),^37^ the M06-class functionals such as M06 and M062X,^38^ and the long-range correction functional (wB97XD).^39^ In all cases, the extended basis set 6-311+g(d,p) including polarization and diffuse functions was used during the molecular optimization.^40^ For solvent model effects a chloroform solution (CHCl_3_) was chosen with solvation model density (SMD)^41^ or polarizable continuum model (PCM).^42^

The results in Table 1 show that B3LYP-GD3BJ with the SMD model solvent is the optimal functional, which reproduces sufficiently the experimental data. Compared with the highest occupied molecular orbital (HOMO) value, the error of the lowest unoccupied molecular orbital (LUMO) value is noticeably larger than that determined experimentally due to the generally greater difficulty of calculating unoccupied orbitals.^43^ Accordingly, the B3LYP-GD3BJ/6-311+g(d,p) level of theory at CHCl_3_ with SMD solvent model (SMD-CHCl_3_) was selected for optimization of all designed structures.

**Table 1:** Theoretical and Experimental *E*_HOMO_, *E*_LUMO_, and Gap Energy *E*_gap_ of the Reference Molecule at the 6-311+g(d,p) Basis Set in CHCl_3_ Solution Using PCM and SMD Model Solvents

	PCM			SMD		
	HOMO	LUMO	*E*_g_	HOMO	LUMO	*E*_g_
B3LYP	–5.39	–3.33	2.06	–5.26	–3.20	2.06
B3LYP-GD3	–5.45	–3.31	2.14	–5.34	–3.16	2.18
B3LYP-GD3BJ	–5.32	–3.35	1.97	–5.19	–3.22	1.97
CAM-B3LYP	–6.79	–2.13	4.66	–6.62	–2.05	4.57
M06	–5.63	–3.16	2.47	–5.51	–3.04	2.47
M062X	–6.55	–2.47	4.08	–6.48	–2.32	3.16
wB97XD	–7.44	–1.54	5.9	–7.40	–1.40	6.00
Experimental	–5.22	–3.41	1.81			

**Figure 2:**
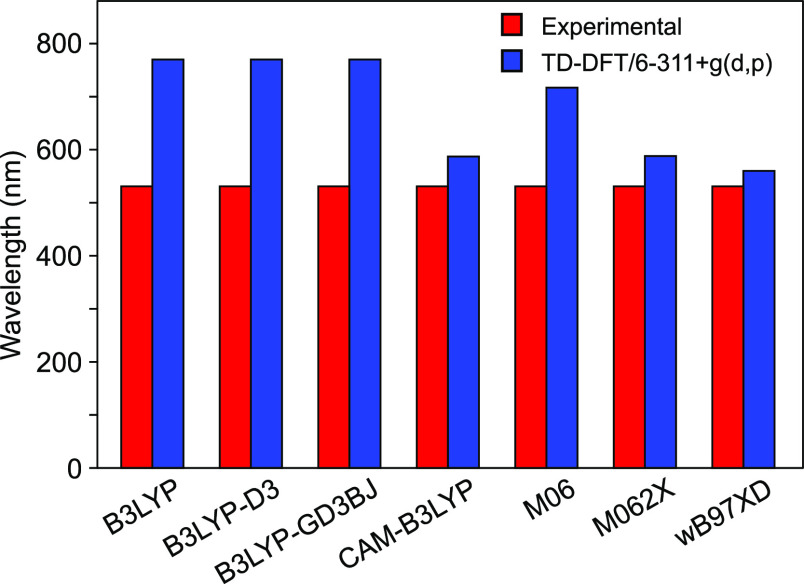
Comparative analysis of experimental and computed maximum absorption wavelengths of DRCN5T with five different functionals. The functional wB97XD is more suitable to reproduce the experimental data.

Next, based on the optimized ground-state structure of DRCN5T at the DFT/B3LYP/6-311+g(d,p) level, TD-DFT calculations were performed using different functionals to theoretically investigate the absorption properties of the reference molecule. Figure 2 presents the simulated spectra that show maximum absorption at wavelength *λ*_max_ of 770, 770, 770, 587, 717, 588, and 560 nm using B3LYP, B3LYP-D3, B3LYP-GD3BJ, CAM-B3LYP, M06, M062X, and wB97XD, respectively. Experimentally, the *λ*_max_ of DRCN5T is found at 531 nm. From Figure 2, the TD-DFT computations based on *λ*_max_ values suggest that wB97XD is the suitable functional to reproduce most accurately the experimental absorption data. Thus, the optical properties of the studied molecules in SMD-CHCl_3_ solution were computed at the TD-DFT/wB97XD/6-311+g(d,p) level of theory.

Subsequently, we calculated hole mobility, which is a crucial parameter of donor materials in organic solar cells. Reorganization energies were calculated based on neutral and cationic states. To determine the hole transfer integrals, we used the M06-2X functional to optimize adjacent molecule pairs and obtain the optimal π-stacking distance. Finally, the donor/acceptor complex, necessary for the evaluation of charge transfer in the organic photovoltaic active layer, was constructed manually out of the selected donor molecule and PC_61_BM as the acceptor molecule. During manual placement, the starting configuration allowed the closest proximity of the molecules while avoiding any steric conflict. Subsequently, we performed an optimization of the donor/PC_61_BM complex using the same previous employed method for molecular optimization (DFT/B3LYP-GD3BJ) to ensure accurate representation of its structure in simulations.

## 3 Results and Discussion

### 3.1 Optimized Structure

**Figure 3:**
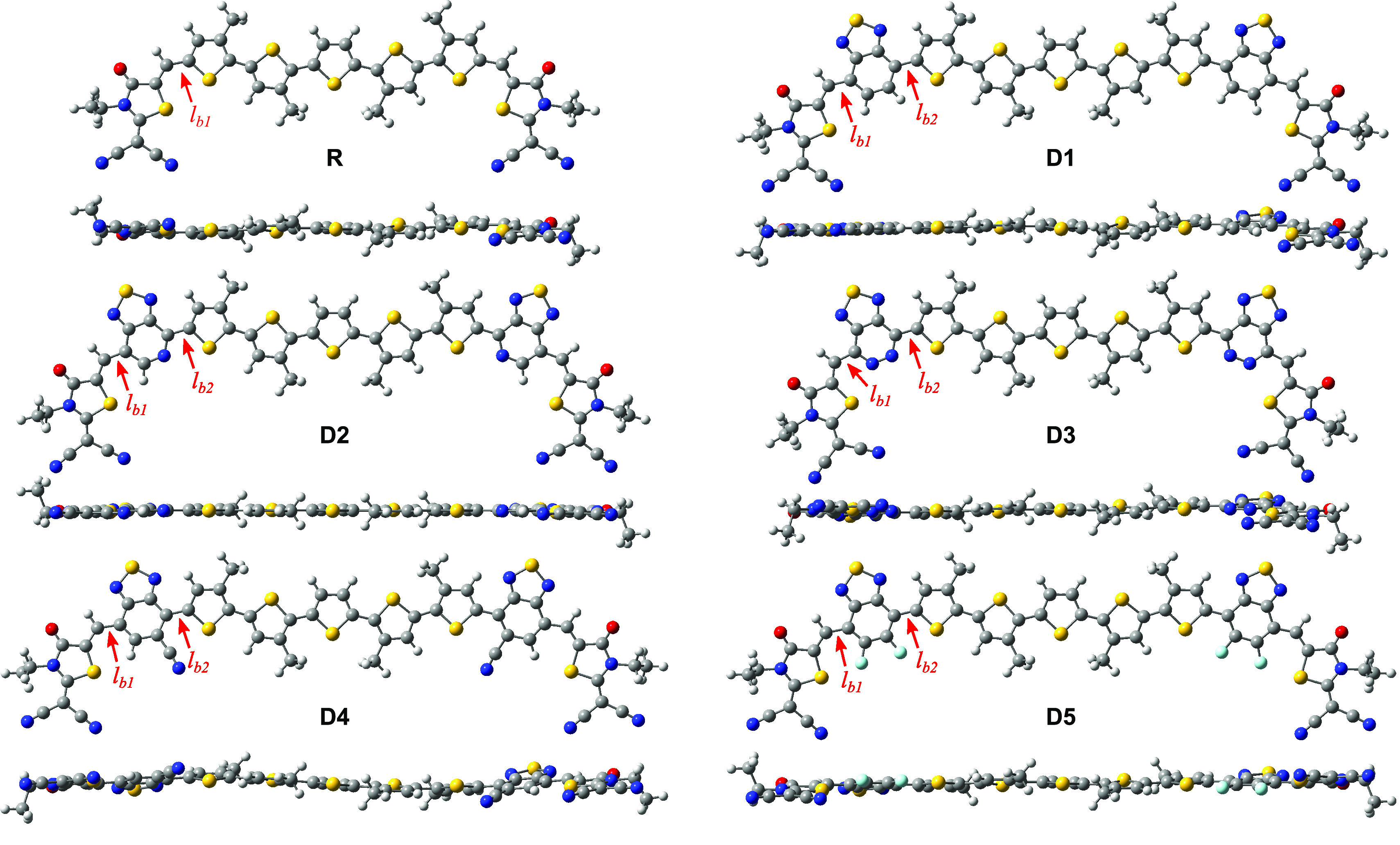
Optimized structures of the studied molecules at the DFT/B3LYP-GD3BJ/6-311+g(d,p) level. The symbols *l*_b1_ and *l*_b2_ denote the acceptor-π-spacer and donor-π-spacer bridge bonds in the modified D1–D5 molecules and the donor–acceptor bond in the reference molecule. Note the large degree of planarity of all considered structures.

Planarity of conjugated materials for OSC applications is necessary as it promotes intramolecular π-orbital overlap and improves the π–π stacking leading to efficient intermolecular interactions.^44^ To ascertain this, ground-state optimizations were performed at B3LYP-GD3BJ/6-311+g(d,p) in chloroform solution. The optimized structures, which exhibit a large degree of planarity, are depicted in Figure 3, and the relevant parameters are tabulated in Table 2. The bridge bond length between the π-spacer and the acceptor (*l*_b1_), between the donor and acceptor in R, and the bridge bond between the donor and the π-spacer (*l*_b2_) have been calculated to gain insight into the electronic interaction strength within the conjugated framework. The calculated bond lengths are found in the range of 1.42 and 1.44 Å, i.e, between the typical C–C single bond length (1.54 Å) and the double bond C=C length (1.33 Å). The short length of these bonds indicates large delocalization of π-electrons in these structures which leads to further intramolecular charge transfer (ICT).^45^

**Table 2:** Parameters of the Optimized Molecular Structures

Compound	*l*_b1_ (Å)	*l*_b2_ (Å)	MPP (Å)	SDP (Å)
**R**	1.42		0.62	3.36
**D1**	1.43	1.44	0.55	3.17
**D2**	1.43	1.43	0.50	3.45
**D3**	1.43	1.43	0.58	3.18
**D4**	1.44	1.44	0.64	3.65
**D5**	1.43	1.44	0.57	3.72

**Figure 4:**
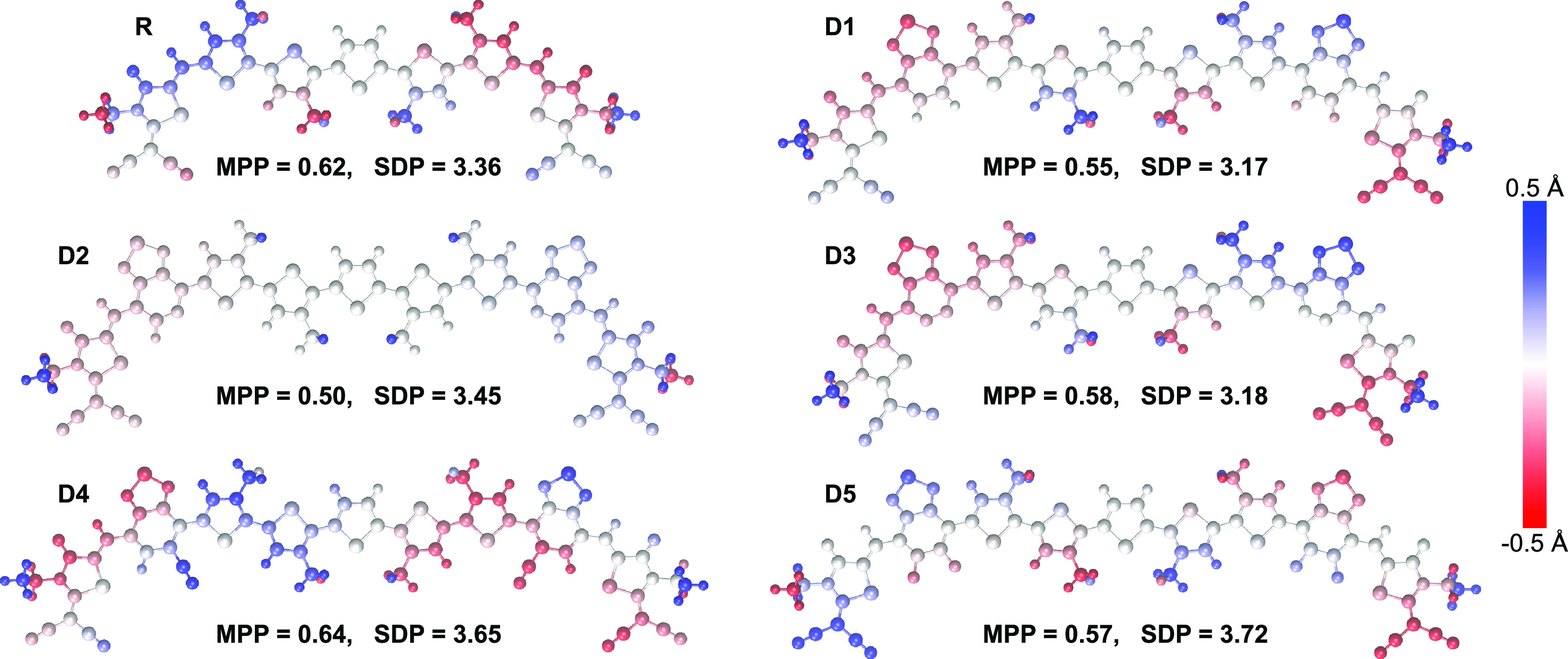
Molecular planarity parameter (MPP) and span of deviation from plane (SDP) plots of R and D1–D5 indicate a large degree of planarity of all considered molecules.

To ascertain the impact of the π-spacer on the overall planarity of the π-conjugated frameworks, the molecular planarity parameter (MPP) and span of deviation from plane (SDP) were calculated using Multiwfn,^46^ and the corresponding structures were plotted using VMD.^47^ The MPP delivers an estimation of the deviation of the whole structure from the plane, while SDP is an indicator of the deviation of different blocks of the structure from planarity.^48^ The low values of MPP and SDP equal approximately 0.6 and 3.5 Å, respectively, denote large planarity of the structures and low deviation from the fitted plane, respectively. A schematic representation of the structures’ deviation is illustrated in Figure 4, with blue/red, respectively, indicating deviation above/below the plane.

The extension of the conjugated framework and the creation of relevant noncovalent interactions (NICs) by adding the π-spacer increase the planarity of the molecular structure. According to Table 2, molecule D2 exhibits the lowest MPP value of 0.50, indicating its superior planarity, which is better than for the reference molecule. In contrast, the D4 π-spacer increases the MPP slightly to 0.64, what is consistent with the largest deviation from planarity for the considered molecules.

The low MPP is likely due to a large conjugated framework and the strong NCIs between the different building blocks. Analyzing the SDP, we notice the out-of-plane deviation of the π-bridge within D4 and D5 structures, which might be explained by steric hindrance^49^ generated by the presence of the electron-withdrawing groups (−CN and −F). However, in general, this validates that the added π-spacer enhances the planarity of the structure, with the small exception of the D4 molecule.

### 3.2 Noncovalent Interaction and Reduced Electron Density Gradient Analysis

To further investigate the designed structures, we evaluated NCIs together with the reduced electron density gradient (RDG). The NCIs-RDG analyses are useful tools to obtain insight into the intermolecular interactions, the repulsive interactions, and the nonlocalized dispersion within the reacting moieties. The RDG is generated from the electron density (ρ)^50^
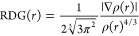
1The RDG scatter graph is generated between RDG versus sign(λ_2_)ρ, where sign(λ_2_) is the second eigenvalue of the electron density (ρ), which is useful to discern the nature of nonbonding interaction, and ρ provides information about the strength of these interactions.^51^

**Figure 5:**
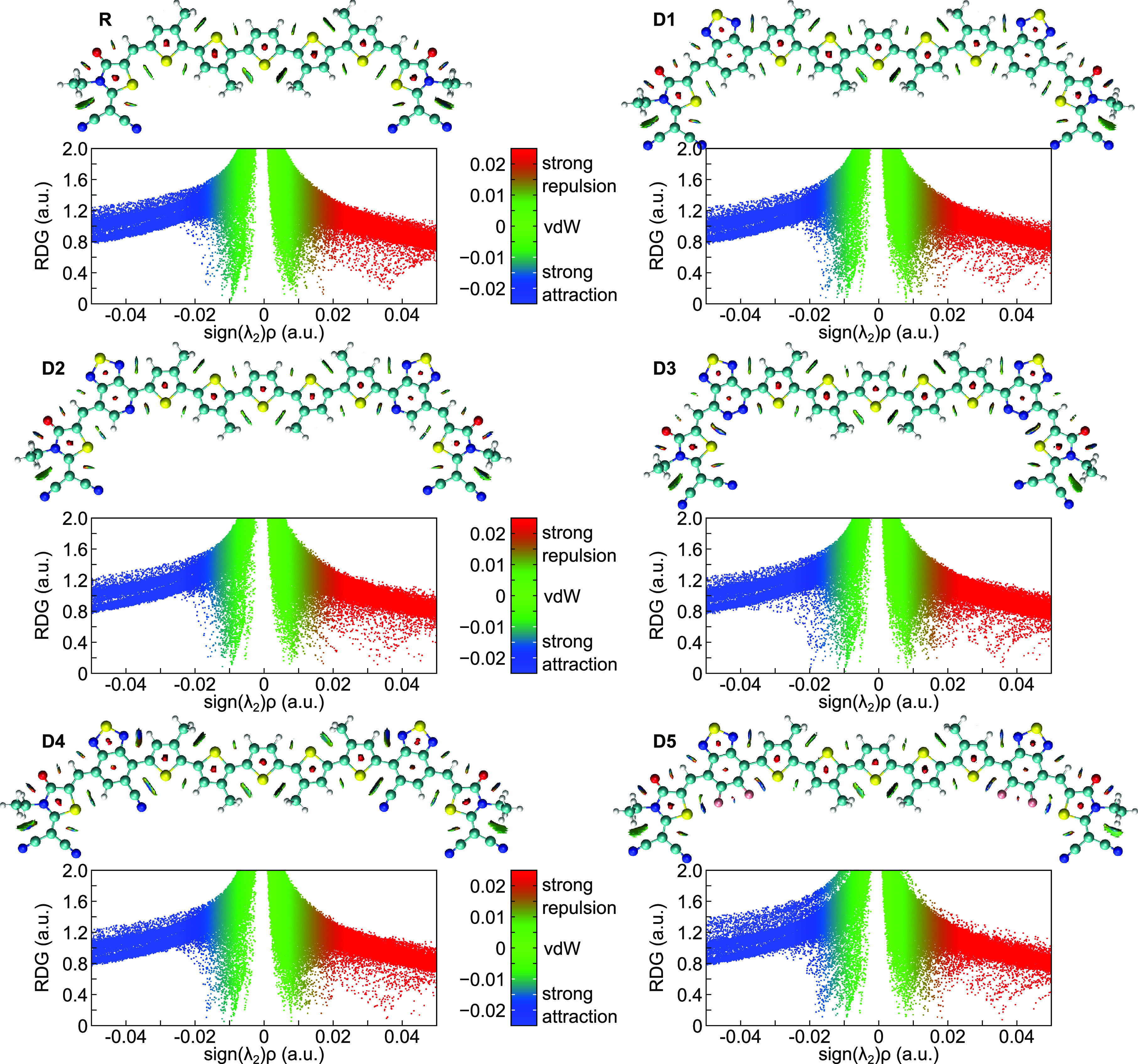
RDG scatter and isosurface plots of R and D1–D5 molecules. The red color indicates the repulsion from aromatic steric effect and the green color indicated the noncovalent interactions.

The graphical illustration of isosurfaces and their respective RDG scatter for D1–D5 and R was performed using Multiwfn and is shown in Figure 5. The value and sign of sign(λ_2_)ρ are interpreted as follows: sign(λ_2_)ρ > 0 defines repulsive interaction from steric effects and is present in aromatic rings and nonbonding interactions, sign(λ_2_)ρ < 0 refers to hydrogen attractive interaction, and sign(λ_2_)ρ around zero corresponds to weak van der Waals interaction.^52^ The vertical color code of RDG scatter spectra, ranging from −0.025 to 0.025 au, presents the λ_2_(*r*) values. The red spikes in the RDG scatter plots between 0.01 and 0.05 au manifest themselves inside the rings of the oligothiophene centered core, the π-spacer, and the acceptor moieties, as shown in the gradient isosurfaces (Figure 5). The blue spikes are weak, confirming the absence of intermolecular hydrogen bonding interactions. The green and mixed red-green spikes observed between −0.02 and 0.01 au indicate the presence of noncovalent interaction between the constructive fragments. As seen from the isosurfaces, the introduction the π-spacer moiety results in relevant intramolecular noncovalent interactions between its ending groups, i.e, hydrogen, nitrogen atoms, the cyano group, and fluorine atoms and the sulfur atoms on the adjacent oligothiophene and acceptor blocks. This “conformational lock” thus leads to an enhanced planarity.^53,54^ The designed materials have shown high planarity generated from the NCIs, which makes them rigid and stable. This proper planarity promotes molecular π–π stacking in the active layer of OSCs.

### 3.3 Frontier Molecular Orbitals

**Figure 6:**
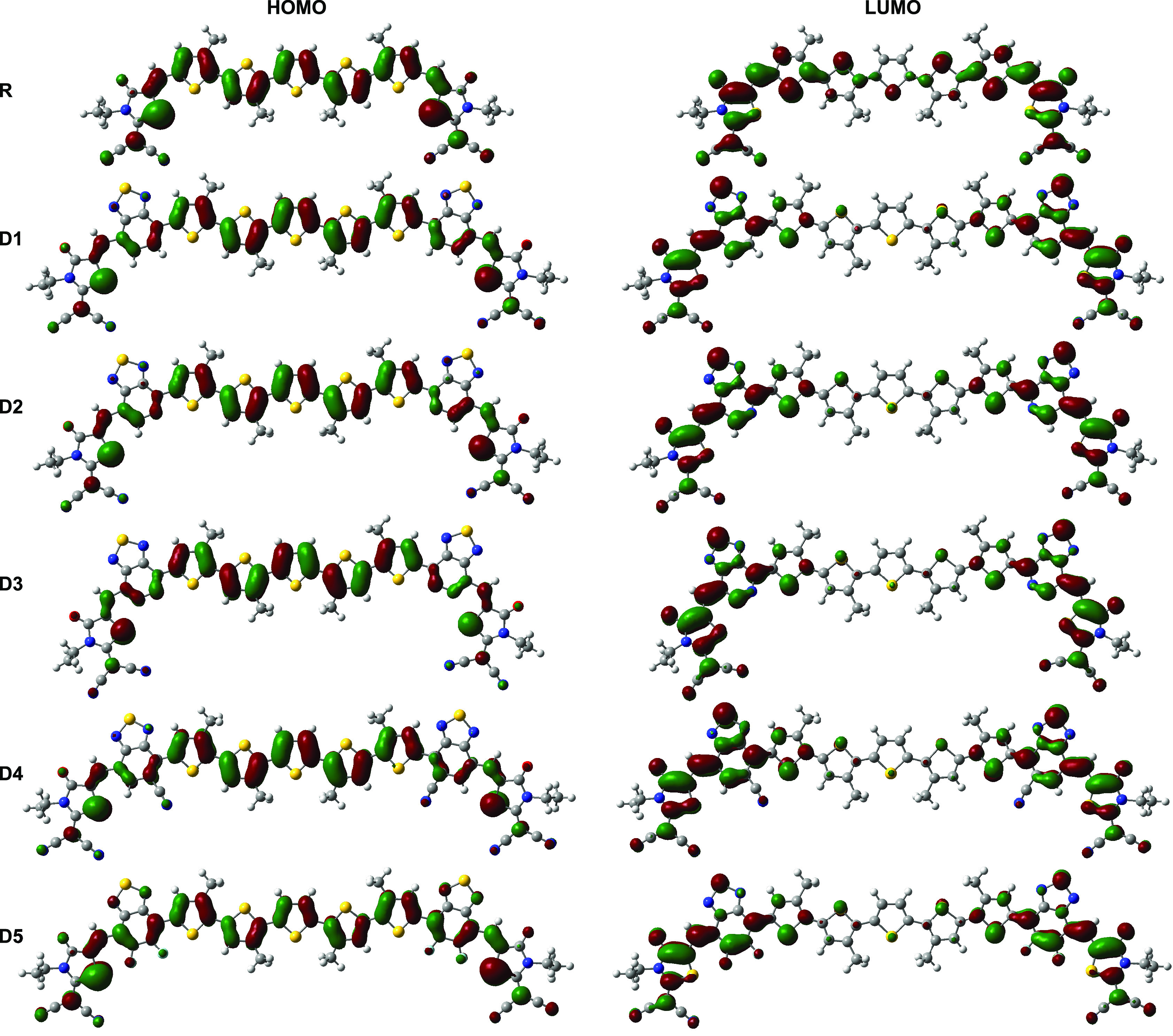
FMO distribution plots of R and designed materials D1–D5. The HOMOs are distributed over the whole structure, while the LUMOs are located over the π-spacer and acceptor, indicating a charge transfer between the building blocks.

**Figure 7:**
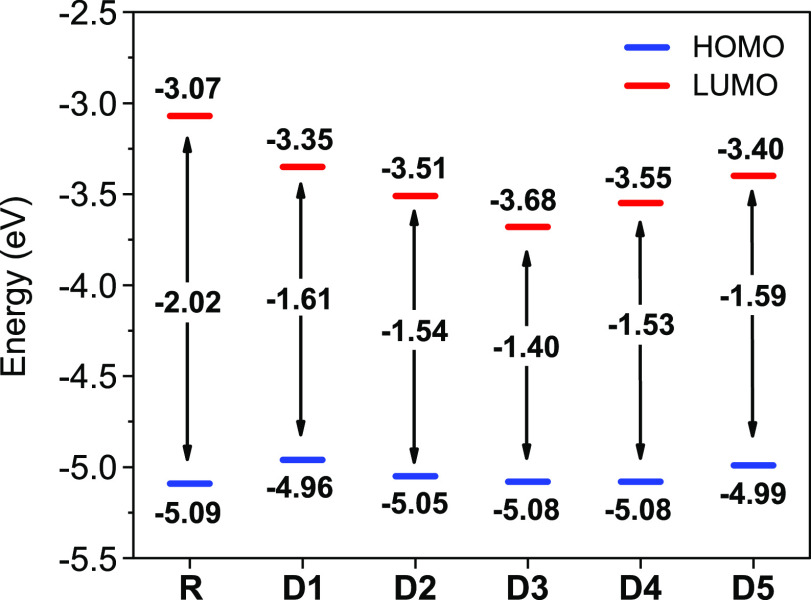
HOMO/LUMO energy levels and band gap energies of R and the designed compound. Note a decrease in the gap energy with the added π-spacer.

The frontier molecular orbitals (FMOs) of conjugated materials are described by the highest occupied and lowest unoccupied molecular orbitals. The FMO analysis is a helpful tool to examine electronic properties and anticipate the optical behavior as well as the ICT within the conjugated backbone.^55^ The HOMO/LUMO charge distributions of the optimized ground-state geometries were performed at the DFT/B3LYP-GD3BJ/6-311+g(d,p) level in SMD-CHCl_3_ solution and are depicted in Figure 6. The HOMOs, LUMOs, and band gap energies are presented in Figure 7 and are listed in Table 3.

**Table 3:** Electronic Properties Calculated at the DFT/B3LYP-GD3BJ/6-311+g(d,p) Level of Theory

Compound	*E*_HOMO–1_ (eV)	*E*_HOMO_ (eV)	*E*_LUMO_ (eV)	*E*_LUMO+1_ (eV)	Δ*E*_gap_ (eV)
**R**	–5.86	–5.19	3.22	–3.02	1.97
**D1**	–5.68	–5.09	–3.52	–3.44	1.57
**D2**	–5.82	–5.16	–3.68	–3.57	1.48
**D3**	–5.89	–5.18	–3.85	–3.75	1.33
**D4**	–5.88	–5.17	–3.70	–3.61	1.47
**D5**	–5.76	–5.14	–3.56	–3.49	1.58

As seen in Figure 6, all of the designed molecules exhibit similar electron density distributions of the HOMO and LUMO states. The HOMOs of R and D1–D5 are characterized by a broad distribution of electron density mainly over the donor moiety. In contrast, the LUMOs are predominantly concentrated over the π-spacer and acceptor moieties. These distributions clearly demonstrate the transport of electrons from the donor over HOMO to the acceptor over LUMO through the π-spacer. The electron-withdrawing nature of the added π-spacer fragments shows its effectiveness for electron migration from the donor to the acceptor.^56^

OSCs require absorber materials with reduced band gaps to maximize their photovoltaic performance.^57^ From Table 3, we note that the designed materials possess smaller band gap energies (*E*_g_) in comparison to R. As seen in Figure 7, the results show a decreasing order of the band gap energies (*E*_g_) as follows: 1.97 eV (R) > 1.58 eV (D5) > 1.57 eV (D1) > 1.48 eV (D2) > 1.47 eV (D4) > 1.33 eV (D3). The fluorine atoms in D5, the cyano group in D4, and the pyridine and pyridazine aromatic rings in D2 and D3 exhibit a larger influence on tuning the electronic properties as compared to the bare benzothiadiazole moiety involving D1. This is related to the high delocalization of electrons and the increased push–pull mechanism.^58^ D3 exhibits the lowest band gap (1.33 eV) resulting from a higher planarity and the high electron-withdrawing behavior of the pyridazine moiety.^59^ These results denote the promising abilities of D1–D5 in OSC applications.

### 3.4 Density of States

**Figure 8:**
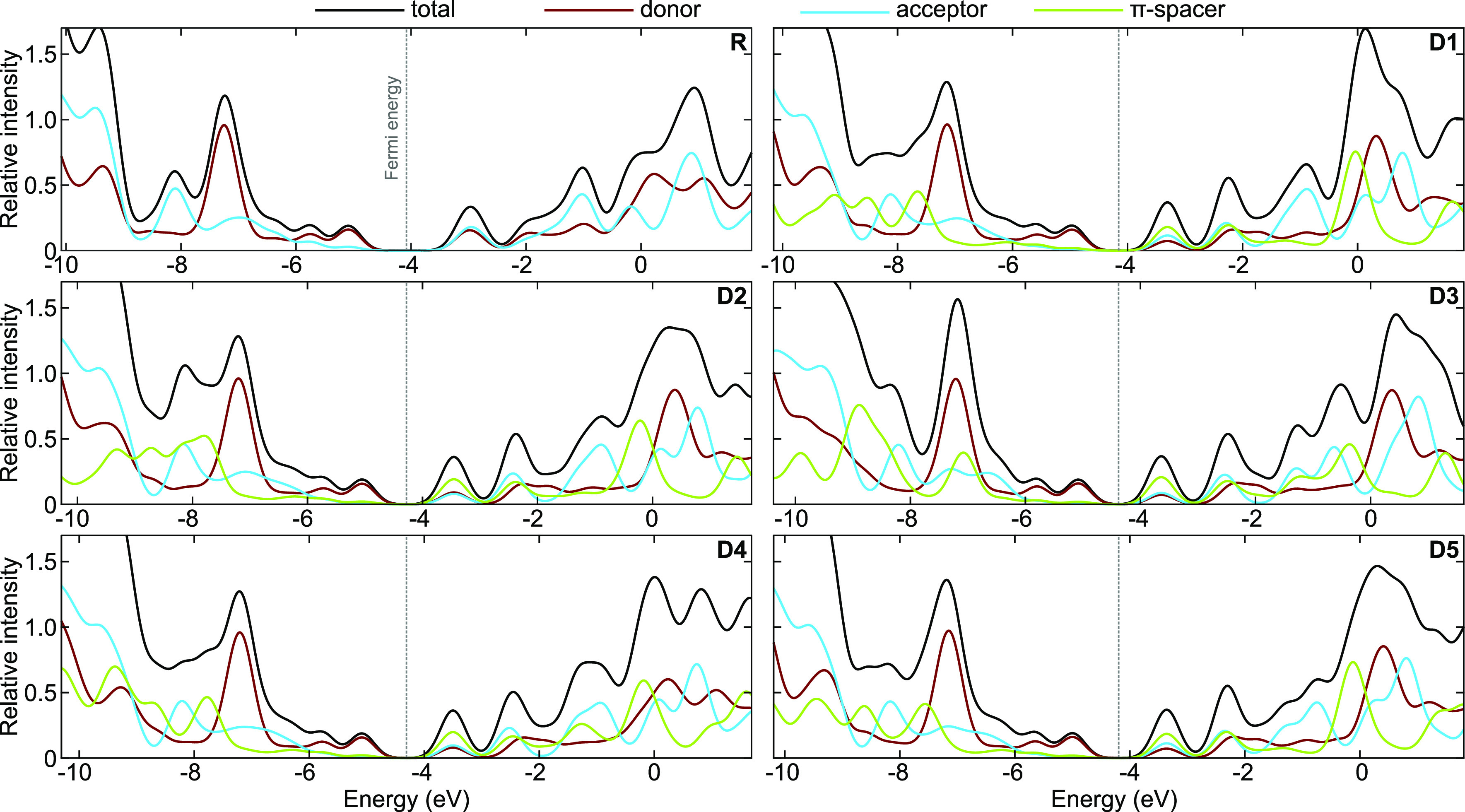
Density of state plots of reference R and designed molecules D1–D5 at the DFT/B3LYP-GD3BJ/6-311+g(d,p) level of theory. The electron density distribution changes while adding π-spacer block which leaded to higher electron delocalization.

The density of states (DOS) provides explicit details on charge occupation and possible electronic excitation over various energetic levels^60^ and is helpful in yielding insight into the contribution of different molecule fragments to the formation of FMOs. Specifically, in Figure 8 we plot both the total DOS (tDOS) of all electrons in the systems and the partial DOS (pDOS) projected onto the three distinct building blocks of the considered molecules (donor, acceptor, and π-spacer) described in Figure 1. The DOS of R and D1–D5, calculated at the B3LYP-GD3BJ/6-311+g(d,p) level of theory, is depicted in Figure 8 and summarized in Table 4. The shape of the tDOS indicates the distribution of electronic energy levels within the molecule. Sharp peaks correspond to localized electronic states, while smooth curves indicate a delocalized electronic structure. In the studied systems, we observe the latter case, i.e., broad tDOS which indicate significant delocalization. In the tDOS curves, the HOMO and LUMO energy levels are easily identifiable. The HOMO levels are located at about −5.0 eV, where distinct peaks in the electron density of states are observed. The LUMOs, on the other hand, appear at around −3.0 eV, which represent the lowest energy level in the region of higher energies. The HOMO/LUMO energy gap, which is approximately 1.9 eV for R and 1.5 eV for D1–D5, is a crucial determinant of the electronic behavior of the molecule, indicating the energy required for electronic transitions.

**Table 4:** Percentage Involvement of Different Segments in Raising FMOs

Compound	FMOs	Donor (%)	π-Spacer (%)	Acceptor (%)
**R**	**H**	84.8		15.2
	**L**	49.8		50.2
**D1**	**H**	88.4	8.4	3.2
	**L**	19.2	50.8	30.0
**D2**	**H**	89.1	7.8	3.1
	**L**	25.0	54.3	20.7
**D3**	**H**	91.3	6.5	2.2
	**L**	18.2	58.8	23.0
**D4**	**H**	89.9	7.9	3.2
	**L**	20.9	57.0	25.6
**D5**	**H**	89.8	7.7	2.5
	**L**	18.8	52.4	28.8

The tDOS plots are divided into three pDOS defining the donor (red), acceptor (blue), and π-spacer (green) moieties of the considered materials, with the band gap clearly visible between the HOMO edge peak on the left and the LUMO level on the right. The strong push–pull interactions between the different fragments are expressed by an increase of the relative peak intensities which enhance the electron density and the electronic transition probability.^61^ Fragments that contribute strongly to HOMO/LUMO formation exhibit a larger electron density, described by large peaks in the DOS plot around the HOMO/LUMO region. These plots clearly show the powerful electron-withdrawing nature of the π-spacing fractions that leads to an alternating distribution arrangement around the HOMO and LUMO levels.

From Table 4, for R, we find a significant 85% contribution of the donor to HOMO with the acceptor providing a minor 15% contribution only. The contributions of donor and acceptor to LUMO are, conversely, equal. These results demonstrate partial electronic density migration from the donor core block to the end-capping acceptor moieties. The added π-spacer changes the electron density distribution of the molecular orbitals and leads to significant electron delocalization and a large charge transfer from the donor to acceptor moieties. Hence, for all of the studied molecules, the HOMO levels are raised mainly due to the influence of the donor. In contrast, the rise in the LUMO levels is a result of the higher percentage contribution of acceptors and bridges. We find that the addition of the π-spacer blocks does not alter the donor contribution to HOMO densities. However, the contribution of the donor to LUMOs decreases considerably (by more than half): from a 50% contribution in R to the range of 20–30% for the modified, with the smallest contribution noted for molecule D2. Due to the π-spacers exhibiting large conjugation and π–π* transition probability, the charge conductivity is higher within the conjugated framework. For all the designed materials, the π-spacer blocks contribute slightly to HOMOs around 8%, while they contribute above 50% for LUMOs. This simultaneously decreases the acceptor contribution to HOMO to 3%. The above discussion proves the role played by the added π-spacer blocks in improving the charge transfer abilities from the core donor to the acceptor moieties.

### 3.5 Optical Properties

**Figure 9:**
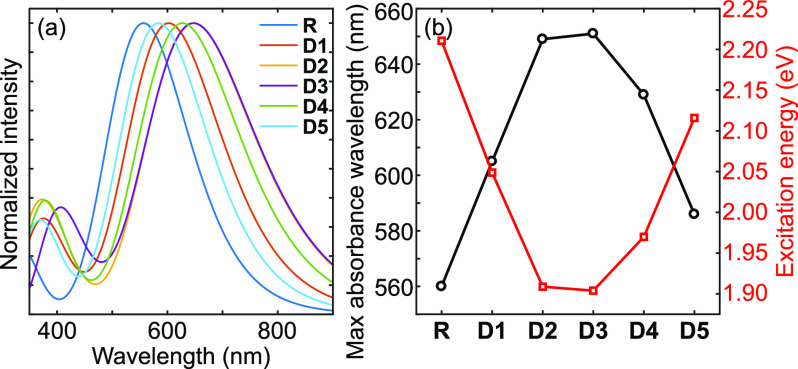
(a) Simulated optical absorption spectra for D1–D5 at the TD-DFT/wB97XD/6-311+g(d,p) level of theory in a CHCl_3_ solution. Note a redshift of the absorption spectra depending on the nature of the π-spacer. (b) Maximum absorption wavelengths and excitation energies of R and D1–D5.

To estimate the optical properties of the studied molecules, TD-DFT was used as a cost-effective method.^62^ The simulated optical absorption spectra were carried out together with the corresponding oscillator strengths at the TD-DFT/wB97XD/6-311+g(d,p) level in SMD-CHCl_3_ solution and are illustrated in Figure 9. The calculated excited energy (*E*_ex_), maximum absorbance wavelength (λ_max_), oscillator strength (*f*), main transitions, full-width at half-maximum (fwhm), and light harvesting efficiency (η_λ_) are tabulated in Table 5.

**Table 5:** Calculated Maximum Absorption Wavelengths λ_max_^abs^ (nm), Electronic Transition Energy *E*_ex_ (eV), Oscillator Strength *f* (au), Full-Width at Half Maximum fwhm (nm), and Main Electronic Transitions

Compound	λ_max_^Exp^(nm)	λ_max_^Theo^(nm)	*E*_ex_ (eV)	*f* (au)	Main transition	fwhm (nm)	η_λ_
**R**	531	560	2.2105	3.1597	H→L (57%)	173.90	0.9993
**D1**		605	2.0488	3.5685	H→L (48%)	216.40	0.9997
**D2**		649	1.9088	3.6260	H→L (55%)	245.27	0.9997
**D3**		651	1.9039	3.0265	H→L (55%)	269.45	0.9990
**D4**		629	1.9699	3.4906	H→L (54%)	236.10	0.9996
**D5**		586	2.1158	3.7290	H→L (50%)	200.92	0.9998

As shown in Figure 9a, the molecules under investigation exhibit large absorption spectra that cover a significant amount of the visible region with a notable red shift compared to that of R. From Table 5, the maximum absorption wavelength (λ_max_) values are found to be 560, 605, 649, 651, 629, and 586 nm for R and D1–D5, respectively, which is in good agreement with the observed trend for *E*_gap_ (Table 3). These λ_max_ values refer to the π → π* electronic transitions involving the electron migration from HOMOs mainly located over the oligothiophene donor unit to LUMOs mainly distributed over the end-capped acceptor and π-spacer moieties.

The λ_max_ values of the designed compounds D1–D5 are, respectively, red-shifted by 45, 89, 91, 69, and 26 nm compared to R. This red-shift indicates a significant contribution of the added π-spacer units to improving intramolecular charge transport (ICT) properties. It originates from the electron-withdrawing groups (−F and −CN) and the acceptor character of the nitrogen atoms within the π-spacer moiety. The red-shift hints at the possible advantage of the light-harvesting ability of the investigated molecules and the improved efficiency of OSCs.

The main contribution to the absorption peaks comes from the HOMO–LUMO electronic transition, as noted in Table 5, showing strong electron displacement from the ground (S_0_) to the first excited state (S_1_). The excitation energy (*E*_ex_) is a key factor in predicting the efficiency of the donor material in OSCs, where *E*_ex_ defines the energy required for an electron to be excited from S_0_ to S_1_. A lower *E*_ex_ is beneficial, leading to easier electronic excitation and smoother charge migration.^63^ This increased ability of molecules D1–D5 to efficiently transport electrons in comparison to R is illustrated in Figure 9b by a significant decrease of *E*_ex_. The excitation energies are larger than the corresponding gap energies because the HOMO → LUMO transition contribution to the main absorption peak is on the order of 50% with additional contributions from weaker transitions from different energy levels. Additional improvement of the photovoltaic performance stems from an increase of the width of the absorption peak, as elucidated by the fwhm (c.f. Table 5). Overall, based on *E*_ex_, the fwhm and a narrow gap of 1.33 eV the D3 molecule is the most appropriate one for the desired electronic application as it exhibits the best optical and charge transport properties due to the existence of a strong electron-accepting entity in the conjugated chain.^64^

The spectral range and intensity of solar absorption are decisive parameters for estimating the short-circuit current density (*J*_SC_) of OSCs. Basically, *J*_SC_ is a function of the external quantum efficiency (EQC) and the photon number *S*(λ) integrated over the solar spectrum, expressed as^65^
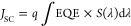
2where EQE is defined as a product of the light harvesting efficiency (*η*_*λ*_), the exciton diffusion efficiency (*η*_ED_), charge separation efficiency (*η*_CS_), and charge collection efficiency (*η*_CC_). The light-harvesting efficiency *η*_*λ*_ depends on the oscillator strength (*f*) of the specific optical absorption wavelength^66^
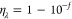
3and together with a broad absorbance is one of the main factors that determines the efficiency of the photovoltaic devices.^67^

The oscillator strength, *f*, is critical in determining the propensity of a donor material to absorb and convert incoming photons into excitations. The value of *f* relies heavily on the choice of functionals, which are used to describe the electronic behavior of the donor material.^68^ From eqs 2 and 3 it is clear that donor materials with large *f* yield high *η*_*λ*_ and provide superior light-harvesting capabilities. As listed in Table 5, *η*_*λ*_ exhibits values close to 1. This convergence is indicative of precise tuning of the density functionals to capture accurately the excitonic behavior of donor materials. In all cases, D1–D5 exhibit larger *η*_*λ*_ compared to R, which is explained by an increased degree of π-conjugation. The obtained results show that all of the designed materials are promising candidates for improving the photocurrent and *J*_SC_ in the OSC devices.

### 3.6 Transition Density Matrix

**Figure 10:**
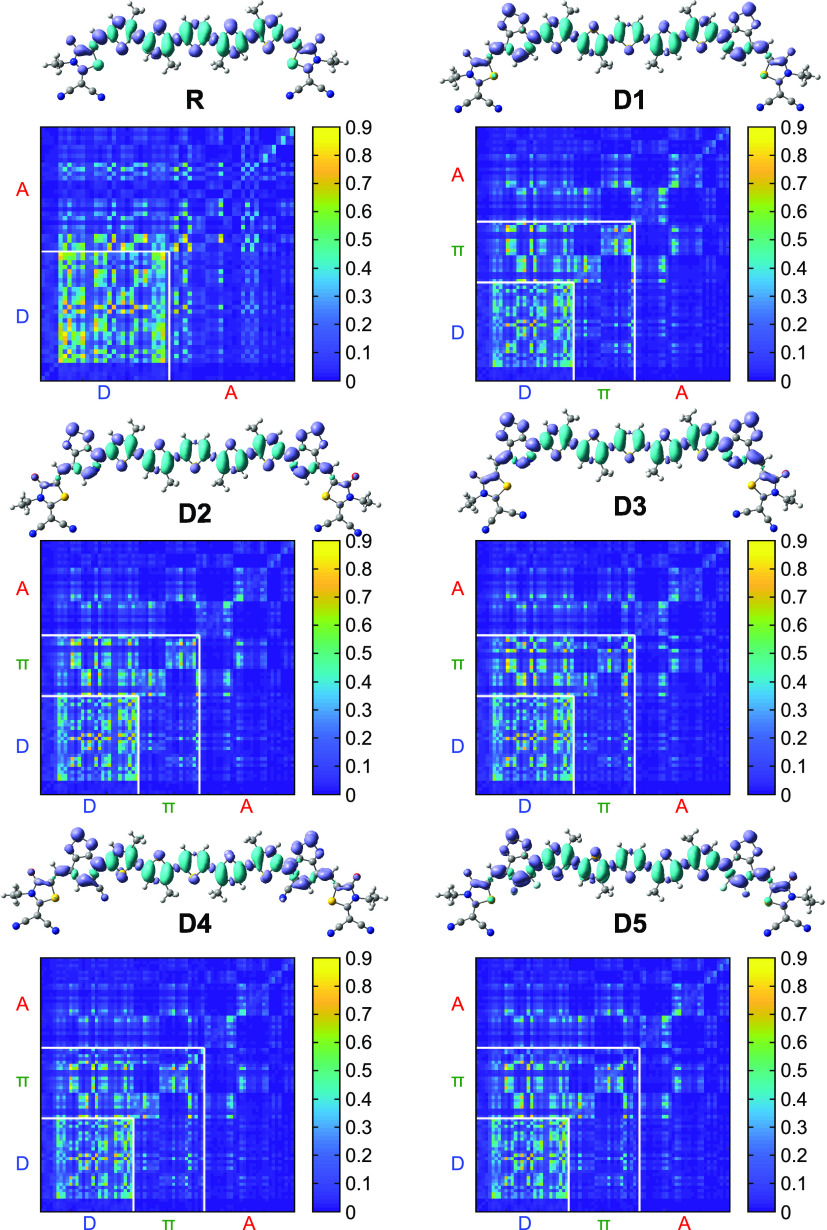
Electron density difference maps and transition density matrix plots of compounds R and D1–D5: Donor (D), Acceptor (A), and π-bridge (π). The charges are dispersed over on-diagonal and off-diagonal segments for D1–D5 compared to R showing enhanced exciton dissociation and higher ICT.

The transition density matrix (TDM) is a useful tool to analyze electronic excitations, electron–hole localization, and interactions between donor, π-spacer, and acceptor moieties. Using Multiwfn, we performed a TDM analysis of the investigated molecules at the first exited state (S_1_) to quantify its composition, identify the atoms most affected by electron transition, and evaluate the hole–electron coherence during the electronic transition.^69,70^ As shown in Figure 10, we divided the TDM maps into three parts representing the different moieties (A, D, and π-spacer) of the conjugated frameworks with the colorbar denoting the electron density coefficient values. Locally excited (LE) state components are marked by the bright diagonal parts, while the off-diagonal elements represent the intramolecular charge-transfer (ICT) state components.

The TMD of the reference molecule shows large electron–hole coherence with the pair localized in the D–D block, indicating the predominance of the local state. A very weak ICT is present between the donor and acceptor elements within R. However, the TDM maps of D1–D5 show a dispersal of charges over the on-diagonal and off-diagonal segments, showing effective exciton dissociation and significant ICT from the donor to the acceptor and the π-spacer elements as compared to R. In fact, the efficient separation of excitons within the donor materials leads to an increase of photogenerated charge carriers and thus improves *J*_SC._(71) The weakest coherence is noted for D4, which contains a strong electron-withdrawing group (−CN) leading to effective exciton dissociation.

Subsequently, we calculated electron density difference (EDD) plots^72^ between S_0_ and S_1_ to study the ICT and charge separation in these materials after electronic excitation. The blue and purple colors of the EDD maps represent the regions of decreasing and increasing electron densities due to electron excitation, respectively. As seen in Figure 10, the donor unit exhibits the minimum electron density, while the π-spacer and acceptor units exhibit the maximum electron density. The decrease of the electron density over the donor is larger for the modified molecules than for R. Simultaneously, the addition of the π-spacer causes a smaller decrease of the electron density over the acceptor unit for D1–D5 compared to R, since the contribution of the acceptor to the HOMO state is reduced by the π-spacer. These plots validate the ICT from the donor-core block to the π-spacer and acceptor blocks during the S0 → S1 transition, demonstrating the contribution of the π-spacer to increasing the electron density difference between the central part and the external part of the molecule and to improving the exciton dissociation into free charges.

### 3.7 Charge Transfer Properties

The charge transfer characteristics of the donor material are used to assess the ability to dissociate excitons into free charges. Following light harvesting in a BHJ solar cell, the excitons created in the active layer at the donor/acceptor interfaces are dissociated into free charges (electrons and holes) with the electrons being injected into the acceptor and the holes being transferred into the donor material to reach the hole transport layer. It is noteworthy that the *J*_SC_ of the solar cell is mainly affected by the efficiency of exciton dissociation and the ability of the active layer to transport charge carriers.^73^ Hence, to ensure good yield of the active layer in the OSCs the donor material should exhibit a large hole transport. The process of hole transfer can be described as a sequence of uncorrelated hopping processes, and the relationship of the hole mobility *μ*_hole_ and the hole transfer rate *k*_hole_ is obtained from
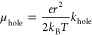
4with *e*, *r*, *k*_B_, and *T* being the electron charge, the intermolecular distance between the π-stacked molecules, the Boltzmann constant, and the temperature (298 K), respectively.

**Figure 11:**
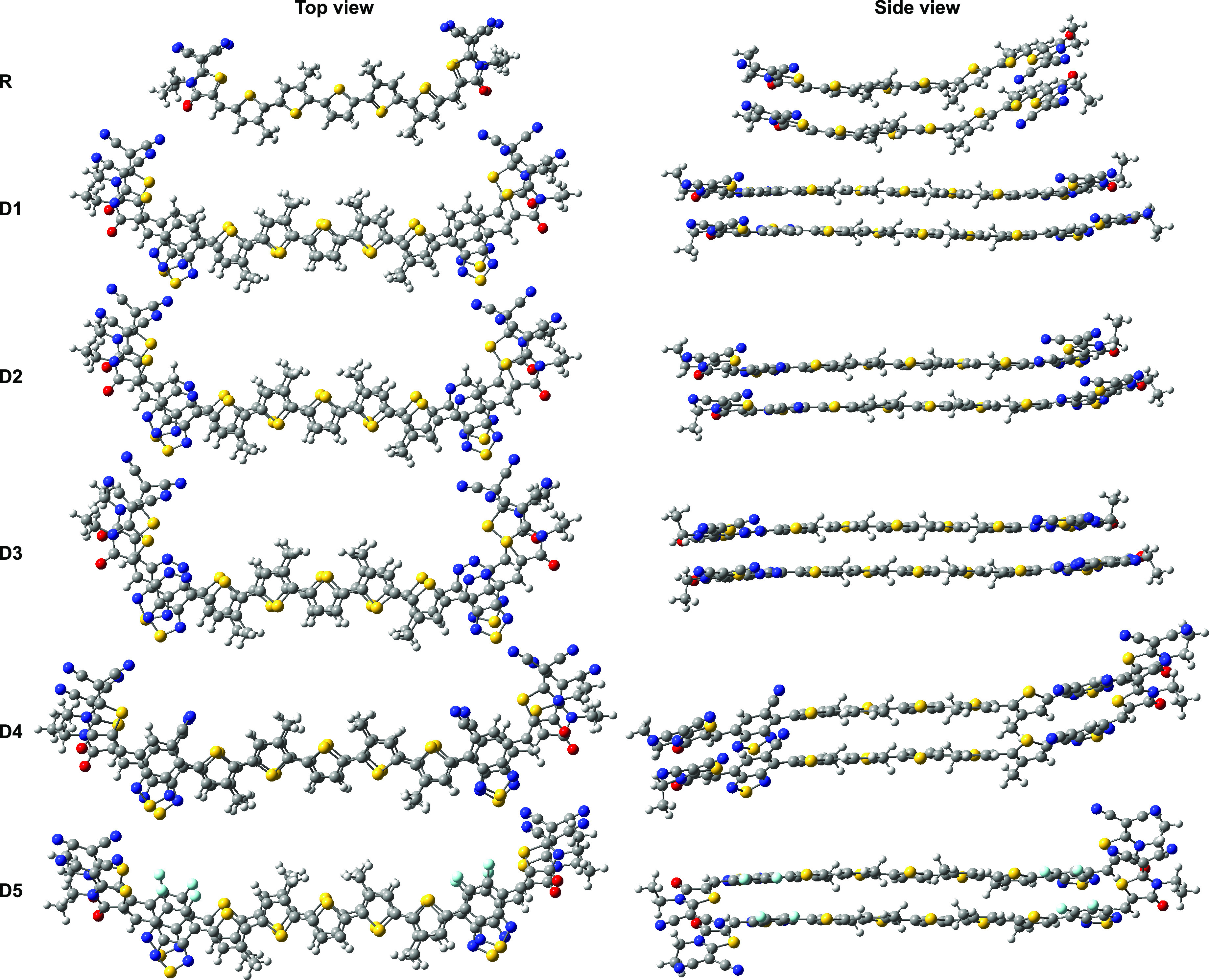
Optimized geometries of the π-stacked configurations of the considered reference and modified molecules at the M06-2*X*/6-31g(d) level of theory.

To obtain insight into these properties, we considered exclusively a face-to-face parallel π-stacking pattern to approximate the charge transport characteristics as it mainly contributes to the process.^74,75^ The M06-2X functional was used to optimize the dimers to obtain the optimal π-stacking distance^76^ at the 6-31g(d) basis set, with the optimized geometries depicted in Figure 11. The center-to-center π-stacking distances are found to be approximately 3.9 Å with a slight perturbation of the structures due to mutual interaction.

**Figure 12:**
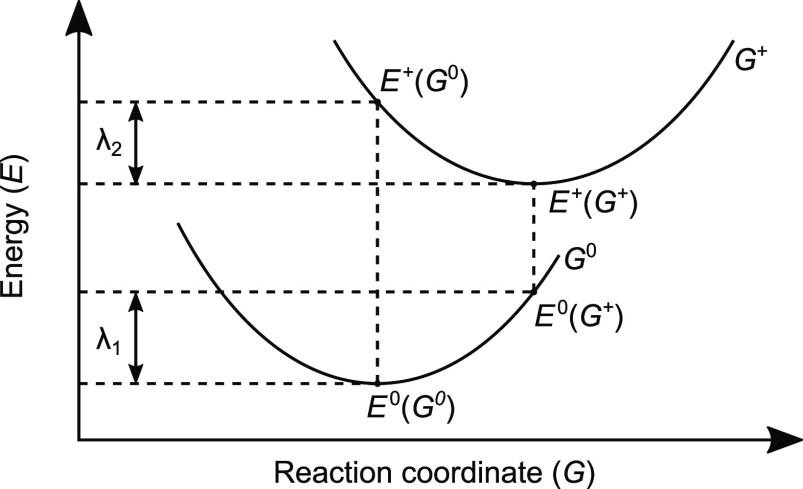
Potential energy curve of the intermolecular transfer reaction between the neutral and cationic states of a conjugated molecule.

The mobility is directly related to the hole transport rate between the neighboring molecules and is calculated based on Marcus theory^77^
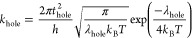
5where *h* is the Planck constant and the temperature is assumed to be 298 K. The relevant parameters in eq 5 for estimating the hole transport abilities are the hole transfer integral (*t*_hole_) and the reorganization energy of the hole (*λ*_hole_). The transfer integral represents the electron coupling strength of the adjacent molecules. According to the Marcus–Hush two-state model, *t*_hole_ is approximated as^78,79^
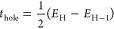
6Here, *E*_H–1_ and *E*_H_ describe the HOMO–1 and HOMO energies of adjacent molecules in the neutral state, respectively. The reorganization energies of holes *λ*_hole_ are calculated based on the neutral cationic states following^80,81^
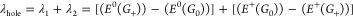
7where *E*^0^(*G*_0_) and *E*^+^(*G*_+_) represent the energies of the neutral and cationic species in their lowest-energy geometries, respectively. Likewise, *E*^0^(*G*_+_) and *E*^+^(*G*_0_) are, respectively, the energies of the neutral and cationic states with the geometries of the cationic and neutral species. As sketched in Figure 12, obtaining the reorganization energies involves four calculations at a single point. The neutral reorganization energy (λ_1_) is equivalent to the difference between the neutral energies of the optimized neutral and charged geometries, respectively. The cation reorganization energy (λ_2_) is equivalent to the energy difference between the cation energies of the optimized charged and neutral geometries, respectively.^82^

From Table 6, the similarity of the reorganization energies of the newly designed materials is explained by the similar relaxation of their geometries. The highest value of *λ*_hole_ found for D5 is likely caused by the presence of electronegative fluorine groups in the π-spacer, which enhance structural relaxation. Molecules with substitutions of −CN and −F show higher hole transfer integrals. This increase in *t*_hole_ is attributed to the electron-withdrawing properties of the fluorine (−F) atoms and the cyano (−CN) groups within the conjugated frameworks. These electron-withdrawing groups promote better stacking between conjugated molecules and improve electronic coupling. The increasing trend of *k*_hole_ (D1 < D2 < D3) is related to the increasing number of nitrogen atoms substituted into the benzene ring of the BT block from zero (D1) to two (D3). This increase leads to a rise of the electron deficiency of the π-spacer and thus enhances electron movement within the conjugated backbone. The *μ*_hole_ goes in order of R < D5 < D1 < D2 < D4 < D3. The high *μ*_hole_ value of 5.53 cm^2^ V^–1^ s^–1^ is found for D3, which shows a low reorganization energy and high intermolecular interactions. In conclusion, these results indicate that incorporating electron-withdrawing groups into a conjugated compound improves its ability to efficiently transport holes. Therefore, according to hole transport mobility calculations, the newly designed materials are expected to exhibit higher transport capabilities, potentially leading to higher values of *J*_SC_ in OSC applications.

**Table 6:** Calculated Reorganization Energies of Hole (*λ*_hole_), Hole Transfer Integrals (*t*_hole_), Hole Transport Rates (*k*_hole_), and Hole Mobility (*μ*_hole_) of R and D1–D5 Studied Materials

Compound	λ_hole_ (eV)	*t*_hole_ (eV)	*k*_hole_ (s^–1^)	*r* (Å)	μ_hole_ (cm^2^ V^–1^ s^–1^)
**R**	0.231	0.13	6.25 × 10^13^	3.88	1.83
**D1**	0.244	0.17	9.54 × 10^13^	3.89	2.78
**D2**	0.202	0.18	1.69 × 10^14^	3.89	4.97
**D3**	0.202	0.19	1.88 × 10^14^	3.89	5.53
**D4**	0.221	0.21	1.82 × 10^14^	3.93	5.47
**D5**	0.267	0.20	9.68 × 10^13^	3.87	2.82

### 3.8 Photovoltaic Properties

Bulk heterojunction solar cells are typically composed of a mixture of π-conjugated electron donor material and a fullerene derivative as the electron acceptor material: (6,6)-Phenyl-C61 Butyric Acid Methyl Ester (PC_61_BM). The power conversion efficiency (PCE) is an important parameter that captures the efficiency of photovoltaic devices and is defined as the ratio of the electrical output to the incident solar power (*P*_in_)^83^
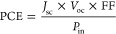
8where *J*_sc_, *V*_oc_, and FF are the short-circuit current density, the open circuit voltage, and the fill factor, respectively. The FF and *V*_oc_ values can be theoretically determined based on the computed electronic properties. To achieve high PCE, the donor should exhibit large FF and *V*_oc_ values, which results in a trade-off for materials with a low energy gap to cover a large area of the solar spectrum. FF is estimated as^84^
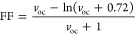
9with *v*_oc_ denoting the dimensionless voltage
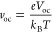
10and *V*_oc_ being^85^
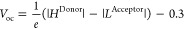
11Here 0.3 is an empirical factor and *H*^Donor^ and *L*^Acceptor^ define the HOMO of the donor and the LUMO of the acceptor, respectively.

**Figure 13:**
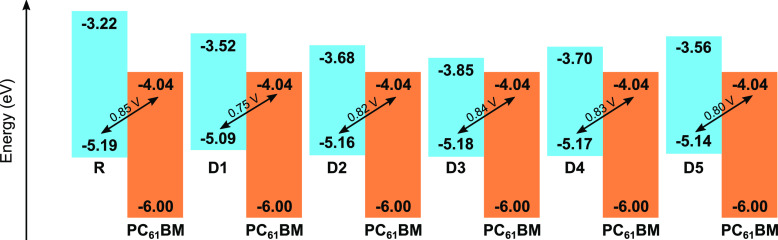
Graphical representation of open-circuit voltage (*V*_oc_) of reference and designed molecules with respect to the acceptor PC_61_BM.

The computed FF and *V*_oc_ of the studied materials are tabulated in Table 7. The investigated donor materials’ HOMO aligned with the LUMO of the PC_61_BM acceptor, along with the *V*_oc_ values are depicted in Figure 13. The *V*_oc_, depicted with the arrows in Figure 13, represents the maximum voltage that an OSC can provide to an external circuit after exciton dissociation. Interestingly, the designed materials exhibit *V*_oc_ and FF values comparable to that of R due to the close HOMO values of these materials.

**Table 7:** Photovoltaic Parameters Calculated for the Studied Compounds

Compound	*V*_oc_ (V)	*v*_oc_	FF
**R**	0.85	33.10	0.867
**D1**	0.75	29.21	0.854
**D2**	0.82	31.93	0.864
**D3**	0.84	32.71	0.866
**D4**	0.83	32.32	0.865
**D5**	0.80	31.15	0.861

By combining these results with the results found previously, it may be deduced that integration of the π-spacer does not necessarily lead to improving all of the properties of the conjugated molecule. Specifically, the π-spacer modification studied here improved mainly the optical and charge transfer properties. In contrast, the photovoltaic properties, which are directly related to the HOMO and LUMO levels of the donor material, are enhanced only slightly. However, all the developed molecules with sufficient *V*_oc_ and FF values can be considered as good candidates for an active layer in BHJ OSC devices.

### 3.9 Donor/PC_61_BM Interfacial Charge Transfer

**Figure 14:**
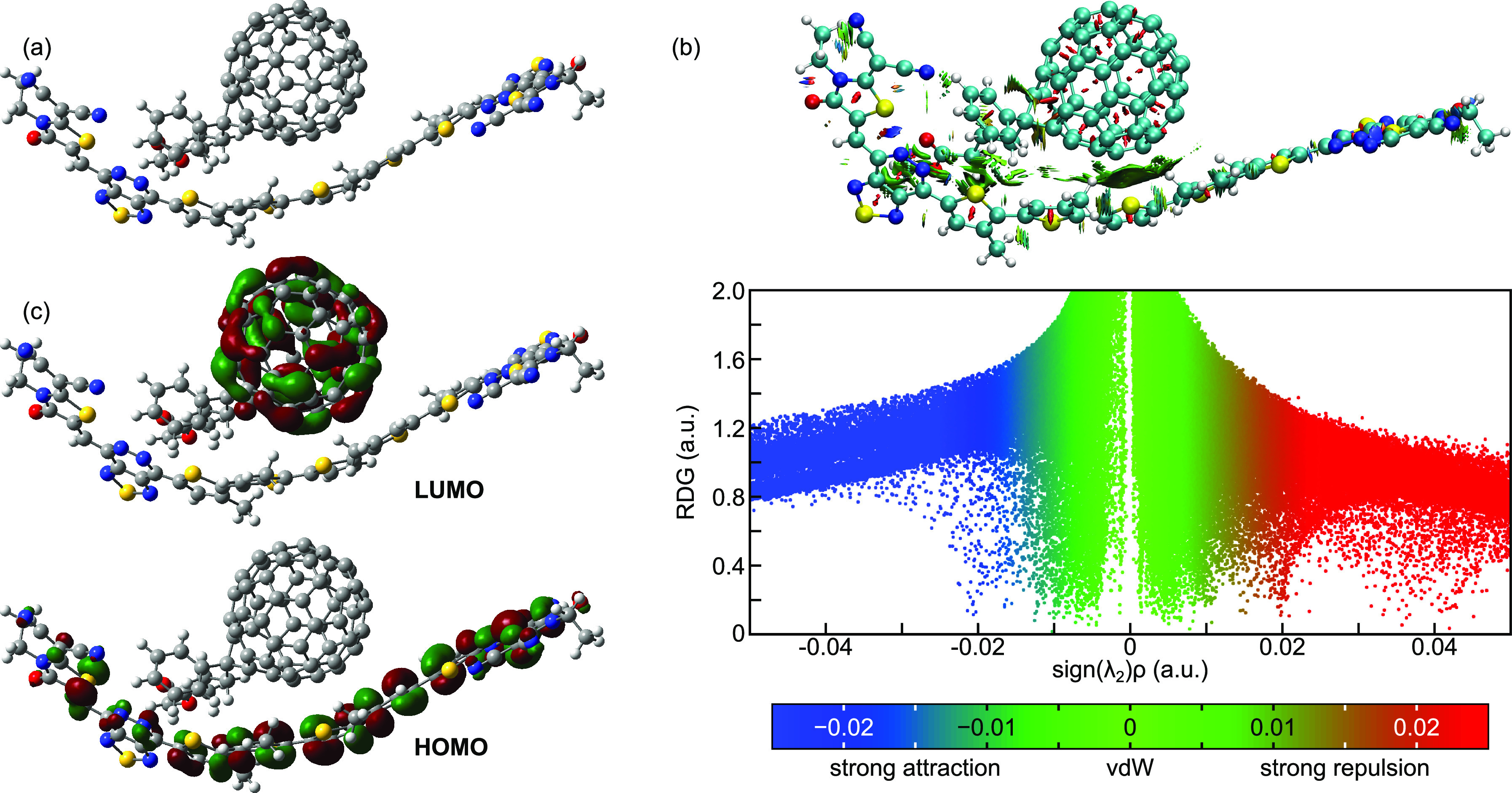
(a) Optimized structure, (b) RDG map, and (c) FMO plots of the D3/PC_61_BM composite.

In order to prove the efficiency of the studied materials as donors, we investigated the charge transfer efficiency of the D3/PC_61_BM composite employing the DFT/B3LYP-GD3BJ/6-31g(d,p) level of theory. The D3 donor is selected due to its superior charge transfer properties, a low hole reorganization energy, and a high hole transport rate. Efficient charge transfer across the interface necessitates that the composite structure maintains planarity and the HOMO/LUMO distribution should be entirely located over the donor and acceptor, respectively.^86^ The optimized D3/PC_61_BM structure, illustrated in Figure 14a, shows that the donor conforms to the acceptor, improving the intermolecular interaction within the composite and facilitating charge transfer within the composite.

In order to ascertain the possible interactions between D3 and PC_61_BM, a reduced density gradient analysis was carried out. As depicted in Figure 14b, high repulsion due to steric effects is located over the aromatic rings of D3 and over PC_61_BM. From the RDG map, the absence of hydrogen bonding is clear. A high degree of van der Waals interaction is seen between D3 and PC_61_BM which favors π–π stacking between the donor and acceptor subparts and thus improves the configuration stability and enhances the ICT. The frontier molecular orbital distribution pattern is illustrated in Figure 14c. Specifically, the HOMO density is entirely distributed over D3, while the LUMO is entirely located over PC_61_BM, demonstrating the donor and acceptor character of these moieties.

## 4 Conclusions

In this study, we report a computational study based on the electronic, optical, and charge transport properties of five novel small molecules to be used as donors in BHJ OSCs by means of DFT. The five molecular structures are derived from the DRCN5T reference by adding benzothiadiazole-derived π-spacer groups to its main framework between the oligothiophene-core donor and the end-caps of the acceptor groups. Considering the obtained results, the addition of π-spacers yields a profound influence on the electronic and absorption characteristics. The [1,2,5]thiadiazolo[3,4-*d*]pyridazine groups (D3) as the π-spacer lead to the largest decrease of gap energies and a red-shift of absorption spectra by increasing the NCIs and enhancing the π-electron delocalization, while the difluorobenzothiazole has a weaker effect. The optical absorption covers most of the visible part of the solar spectrum with a high light-harvesting efficiency. An analysis of the charge transport shows the effect of the π-spacer units on the exciton dissociation at the first excited state and enhancing the charge carrier mobility. The newly designed materials show enhanced properties in all of the studied aspects of OSCs compared to the reference molecule. Due to the considerable electronegativity of the nitrogen atoms within the π-spacer and the high ICT, D3 exhibits the largest maximum absorption wavelength of 651 nm and the largest hole mobility of about 5.35 cm^2^ V^–1^ s^–1^. Accordingly, the D3/PC_61_BM composite was studied to evaluate the charge transfer between the donor and acceptor subparts. Overall, this study shows that adding a π-spacer building blocks to the molecular structure can be a promising strategy to further improve the photovoltaic properties of donor materials for highly efficient OSC devices.

## Data Availability

All data underpinning the results of this study is available from the Authors on reasonable request. Basic data files necessary to reproduce the main results of this study are also available at 10.5281/zenodo.10137672.
